# Wear Behaviour of Multilayer Al-PVD-Coated Polymer Gears

**DOI:** 10.3390/polym14214751

**Published:** 2022-11-05

**Authors:** Tonica Bončina, Brigita Polanec, Franc Zupanič, Srečko Glodež

**Affiliations:** Faculty of Mechanical Engineering, University of Maribor, Smetanova 17, 2000 Maribor, Slovenia

**Keywords:** polymer gears, aluminium PVD coating, multilayer coating, wear

## Abstract

A comprehensive experimental investigation of the wear behaviour of coated spur polymer gears made of POM is performed in this study. Three different thicknesses of aluminium (Al) coatings were investigated and deposited by the Physical Vapour Deposition (PVD) process. The Al coating was deposited in three steps: By plasma activation, metallisation of the aluminium by the magnetron sputtering process, and plasma polymerisation. The wear of the gears was tested on an in-house developed testing rig for different torques (16, 20, and 24 Nm) and a rotational speed of 1000 rpm. The duration of the experiments was set to 13 h, when the tooth thickness and, consequently, the wear of the tooth flank were recorded. The experimental results showed that the influence of metallisation with aluminium surface coatings on the wear behaviour of the analysed polymer gear is not significantly important. The results also showed that the gears with a thicker aluminium coating showed greater wear than gears with a thinner coating or even without a coating. This is probably due to the fact that the aluminium particles that started to deviate during gear operation represented the abrasive material, which led to the faster wear of the contacting surfaces of the meshing gear flanks.

## 1. Introduction

In recent decades, polymer gears have been used widely in many engineering applications (office appliances, mechatronic devices, household facilities, computer and laboratory equipment, medical instruments, etc.) due to some of their advantages over metal gears [1,2,3,4]. Namely, polymer gears have been replacing metal gears because of their financial benefits in mass production, where injection moulding technology [5,6] is often used to produce polymer gears. Furthermore, polymer gears can operate without additional lubrication and, consequently, may be used in applications where a lubricant is not desired (printers, the food industry, medicine, etc.). Some other benefits of polymer gears are low weight, good resistance to corrosion, low coefficient of friction, low noise, the ability to absorb and damp vibration, etc. [7,8,9,10,11]. However, polymer gears also have some disadvantages, such as worse mechanical properties (Young’s modulus can be as much as one hundred times lower than the values obtained for steel), which may also vary significantly with temperature [12,13]. Compared to metal gears, polymer gears have poorer thermal conductivity and temperature resistance, resulting in increased operating temperatures and, consequently, thermal failures (melting) of gear teeth [14,15]. Last but not least, polymer gears have lower manufacturing precision (especially in the case of moulded gears) and relatively high dimensional variations due to temperature and humidity conditions [16,17].

In contrast to metal gears, for which the design procedure and determination of load capacity is standardised with the ISO 6336 Standard [18], it is not valid for polymer gears. Therefore, the VDI 2736 guideline [19] is currently the only practical tool for designing and calculating the load capacity of polymer gears. As already addressed in [19] and explained additionally in [20,21,22], the following failures and their causes are typical for polymer gears: melting, tooth root fracture, pitting of tooth flanks, wear of tooth flanks, and tooth deformation.

The investigation presented in this study is focused mainly on the wear phenomenon on polymer gears. Because polymer gears usually run without lubrication (in dry operating conditions), the high contact friction and, consequently, a high degree of wear typically appear in such cases. The final result is the short service life of such gear drives, especially in high-power transmission applications. Recently, many researchers have been investigating different influencing factors on the wear behaviour of polymer gears. Singh et al. [23] investigated the wear resistance of polymer gears made of ABS, HDPE, and POM at different torques and rotational speeds. Their experimental results showed the highest wear resistance for POM and the lowest for ABS. Li et al. [24] found out that the wear of polymer gears can be reduced significantly by micro geometry modification (tip relief) of gear flanks. Evans et al. [25] and Lin et al. [26] proposed a novel approach to describe the wear behaviour of polymer gears, considering the influence of a given loading and operation speed. Mao et al. [27] showed that the manufacturing process (machine cutting, injection moulding) does not influence the wear behaviour significantly, which means that polymer gears can be produced using injection moulding technology if it becomes more suitable (cheaper) compared to machine-cut polymer gears. Gürgen et al. [28,29] investigated the wear behaviour of Ultra-High Molecular Weight PolyEthylene (UHMWPE) composite reinforced with hard particulate fillers, considering elevated temperatures and the oxidative effect. The authors concluded that the wear behaviour of the composite changed heavily due to the increasing temperatures. On the other hand, oxidation leads to a heavy degradation in the anti-wear properties of the analysed composite.

As proposed by Hoskins et al. [30], polymer gears often operate without lubrication because of special requirements in different applications where lubricants cannot be used (the food industry, medicine, office equipment, etc.). However, a high coefficient of friction and, consequently, a high amount of wear may appear in such cases. One way to reduce the friction of nonlubricated polymer gear pairs is the application of low-frictional coatings [31]. The work published by Dearn et al. [32] showed that the friction and wear of nonlubricated polymer gears can be reduced by using solid lubricant coatings (MoS_2_, PTFE, etc.). The tribological characteristics and, consequently, wear resistance of a common solid lubricant, polytetrafluoroethylene (PTFE), may be improved using a certain nanofiller (Alam et al. [33,34]). Furthermore, Bae et al. [35] proposed a computational approach to obtain the contact stresses of coated polymer gears, considering the influence of the coefficient of friction on the stress field in meshing gear teeth. Their numerical results showed the negligible effect of a 2-µm-thick coating on contact stress because the thickness was insufficient to affect the bulk deformation behaviour of the polymer gears. On the other hand, a thicker coating may also influence the tooth tip deflection of polymer gears. The computational analyses presented by Trobentar et al. [36] showed that the tooth tip deflection decreases with higher values of Young’s modulus of the coating material and a larger thickness of the surface coating.

Physical Vapour Deposition (PVD) is another technique that may be used to improve the wear resistance of contacting mechanical elements [37,38,39]. PVD technology is used widely for the deposition of thin films to improve tribological and optical characteristics in several fields of industry: biomedicine [40,41,42], solar equipment [43], low-friction applications [44], cutting tools [45,46,47], etc. Furthermore, PVD processes allow the deposition of multi-layered coatings, as well as different alloy compositions [48]. Maurer et al. [49] and Coto et al. [50] showed that multilayer titanium coatings increased the erosion resistance of PEEK significantly. However, the deposition of thin PVD coatings on the polymeric substrate is limited due to the low-temperature resistance and bad adhesion ability of polymers. In that respect, the appropriate pre-treatment of the polymeric substrate is often required to improve the adhesion of the surface layer and, consequently, to reach a good performance of the surface coating [51].

In the authors’ previous work [52], we analysed the fatigue behaviour of coated spur polymer gears, where only one coating layer was investigated, made of aluminium (Al), chromium (Cr), or chromium nitride (CrN). The experimental results showed that the influence of Al, Cr, or CrN surface coatings on the wear behaviour of the analysed polymer gear was not significant. This was explained by the fact that the analysed coatings were very thin and, therefore, did not influence the wear resistance significantly. For that reason, an experimental investigation of the wear behaviour of multilayer aluminium PVD-coated spur polymer gears made of POM is presented in this study. Furthermore, an additional adhesion analysis of a multilayer aluminium PVD coating was performed in the framework of the proposed research. The deposition process of the analysed coatings is described briefly in Section 2.1, while the testing procedure is discussed in Section 2.2. In Section 3, the obtained experimental results regarding the adhesion analysis and wear behaviour are summarised and evaluated critically.

## 2. Materials and Methods

### 2.1. Materials

**Figure 1:**
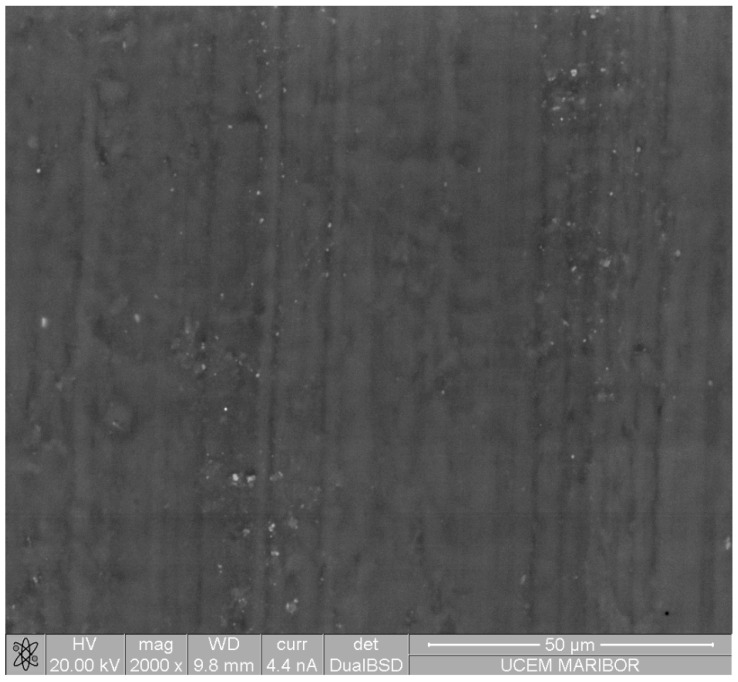
The backscattered electron micrographs of the hobbed gear made of POM.

In this work, the polymer gear specimens made of Polyoxymethylene (POM) were machine cut from extruded bars using a hobbing process. The basic physical and mechanical properties of the used polymeric material are presented in Table 1. The surface appearance of the gears can be seen in Figure 1.

### 2.2. Methods

#### 2.2.1. Coating Deposition Process

In the presented study, the PVD process was used to apply pure aluminium multilayer coatings on the POM substrate. The aluminium coating was applied through a plasma activation process, followed by metallisation of the aluminium through a magnetron sputtering process, and, finally, a plasma polymerisation step.

The samples were sputtered on a META ROT 500 machine with a horizontal short-cycle system for metallisation, including a part for sputtering protective coatings. In this machine, the tool with the loaded samples is inserted into the device before the process starts. When the door is closed, pumping starts in pre-vacuum mode. When a certain vacuum is reached, pumping is switched to a more powerful, high-vacuum mode (pumping takes place via diffusion pumps and, additionally, with water molecules, using the Polycold cryopump). The first process step, plasma activation, is started after reaching the initial pressure (5 × 10^−3^ mbar). The MFC (Mass Flow Controller) begins to supply 800 sccm (standard cubic centimetre per minute) of air (77% nitrogen) to the chamber. After the stabilisation time of 6 s has passed, the plasma generator is switched on. The power reaches 12 kW in 3 s. After 15 s, it switches off at this intensity.

The next process step is the aluminium sputtering. The process is carried out in a high vacuum, using argon plasma gas. The argon atoms accelerate in the plasma field and collide with the aluminium target. The knocked-out aluminium atoms condense to polymer samples, which are attached to a rotating planetarium. The planetary gear rotates at 50 rpm during the process. Temperature (min 30 °C, max 90 °C) refers to the limit temperature for the target or magnetron. The magnetron is cooled with warm water during the process, which prevents the excessive contraction and expansion of the target. During the process, the samples are heated to approximately 60–80 °C due to process influences.

The last process step is plasma polymerisation. It is carried out in a fine (pre)vacuum by pumping the pre-pumps only so as not to contaminate the oil in the diffusion pumps. HMDSO (hexamethyldisiloxane) is fed into the chamber and condensed on the samples. The HMDSO is polymerised in the plasma field on the samples and forms a thin protective layer on the aluminium that prevents oxidation and other atmosphere influence.

The coated polymer gears with 1, 3, and 5 layers were tested and analysed in the presented study (see Section 3). The process parameters for one layer of analysed aluminium coating are shown in Table 2. In the case of multilayer coatings, each process step as described above was repeated 3 times for 3-layer coating and 5 times for the 5-layer coating.

#### 2.2.2. Coating Characterisation

The surface of the uncoated polymer substrate was examined using the Environmental Scanning Electron Microscope (Quanta 200 3D, FEI, Eindhoven, The Netherlands), which allows analysis of electrically and thermally non-conductive materials. The pressure of water vapour in the chamber was 60 Pa, and two detectors were used for imaging: an LFD (Large Field Detector) for secondary electrons and a Backscattered Electron Detector (BSE).

The surfaces of the coated samples were analysed using a high-resolution Scanning Electron Microscope (Sirion 400 NC, FEI, Eindhoven, The Netherlands) equipped with an Energy Dispersive Spectrometer (INCA x-sight, Oxford Analytical, Bicester, UK). The EDS was taken at 5 kV of accelerating voltage in order to reduce the interaction volume. A Focussed Ion Beam installed in a Scanning Electron Microscope (SEM-FIB; Quanta 200 3D, FEI, Eindhoven, The Netherlands) was used to prepare cross-sections through the coatings. The primary opening (required for further observations of the cross-sections) with dimensions 20 × 10 × 5 µm was ion milled with Ga-ions at 30 kV accelerating voltage and a 1 nA beam current. After milling, the specimen was tilted, and the image of the cross-section was taken using low ion beam currents of 10 and 30 pA at the same accelerating voltage.

#### 2.2.3. Indentation Tests and Adhesion Analysis

Indentation tests were used to measure the indentation hardness and indentation modulus. The equipment used in this study was a Nano Test Vantage (Micro Materials Limited, Wrexham, UK), which was equipped with a Berkovich diamond indenter. The indentation instrument was controlled by an electromagnetic drive loading system with a high-precision coil and a permanent magnet. The tests were carried out with increasing loads. A test series was carried on the low load head (maximum load 500 mN) from 1 to 5 mN at the same place. The loading time was 200 s while holding at the maximum load and the unloading time was 10 s. The indentation curves were corrected for the thermal drift, and the diamond area function *A* = 500 *h* + 23.5 *h*^2^ was used for the calculation of the indentation properties.

Adhesion analysis was performed using a scratch test. The diameter of the spherical diamond tip was 23 µm. At each test, five scratches were made using the scanning velocity of 10 µm/s. A very low load of 0.1 mN was used for the first 50 µm. For the next 250 µm, the load increased with a rate of 10 mN/s up to the maximum load of 250 mN. The constant maximum load was then applied for the last 50 µm of scratch. The surface topography was measured before and after the test, and then the initial roughness was subtracted from the measured topography during and after the test. The damaged surface was evaluated using SEM micrographs, and the critical load for causing damage was determined, similar to that defined by the ASTM C1624 [54] and EN ISO 20502:2016 [55] standards.

#### 2.2.4. Gear-Wear Tests

**Figure 2:**
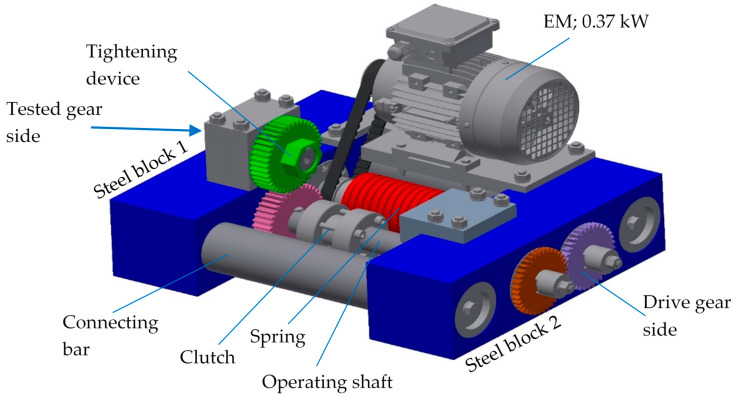
The prototype of the gear test rig.

**Figure 3:**
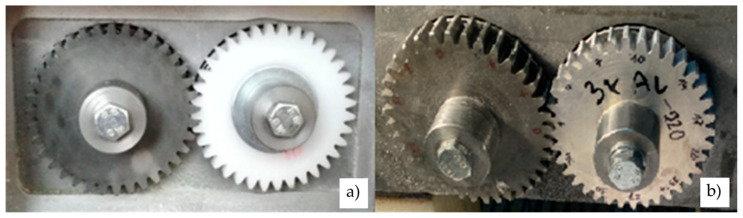
Tested polymer gear made of POM: (**a**) Without coating, (**b**) Al coating.

The gears were tested on an in-house developed testing rig, as shown in Figure 2. The test rig employs the general “back to back” principle, which is often used for testing metal gears. The closed-loop consists primarily of two operating shafts connected with two gear pairs: the drive gear pair (both gears were made of steel) and the tested gear pair (the tested gears were made of POM, and the supported gear was made of steel). The tested gear pair is shown separately in Figure 3, while the basic geometric parameters of the tested gear pair are presented in Table 3.

The testing procedure followed the general guidelines for testing polymer gears as described in VDI 2736 [19]. Before the test began, the tested polymer gears were weighed on a technical balance, Mettler Toledo AX 204 SI 01 05 02, with a weighing accuracy of up to 0.1 mg. Furthermore, the tooth thickness was measured using a Mitutoyo Absolute dial gauge with a roller diameter of 5 mm. The rotational speed was set to 1000 rpm and was controlled using a Voltcraft DT-10L strobe. The experimental testing was performed for the torques 16, 20, and 24 Nm. The duration of the experiments was set to 13 h when the tooth thickness and, consequently, the wear of the tooth flank were recorded. Up to three tests for each loading configuration were then considered when presenting the experimental results (see Section 3).

## 3. Results and Discussion

### 3.1. Coating Characterisation

Table 4 gives the indentation hardness and reduced modulus for both uncoated and coated samples, as well as for pure Al. The results show a very small increase in both properties due to the coatings. The indentation hardness and reduced modulus increased with the increasing thickness of coatings and were much lower than the known properties of pure aluminium.

**Figure 4:**
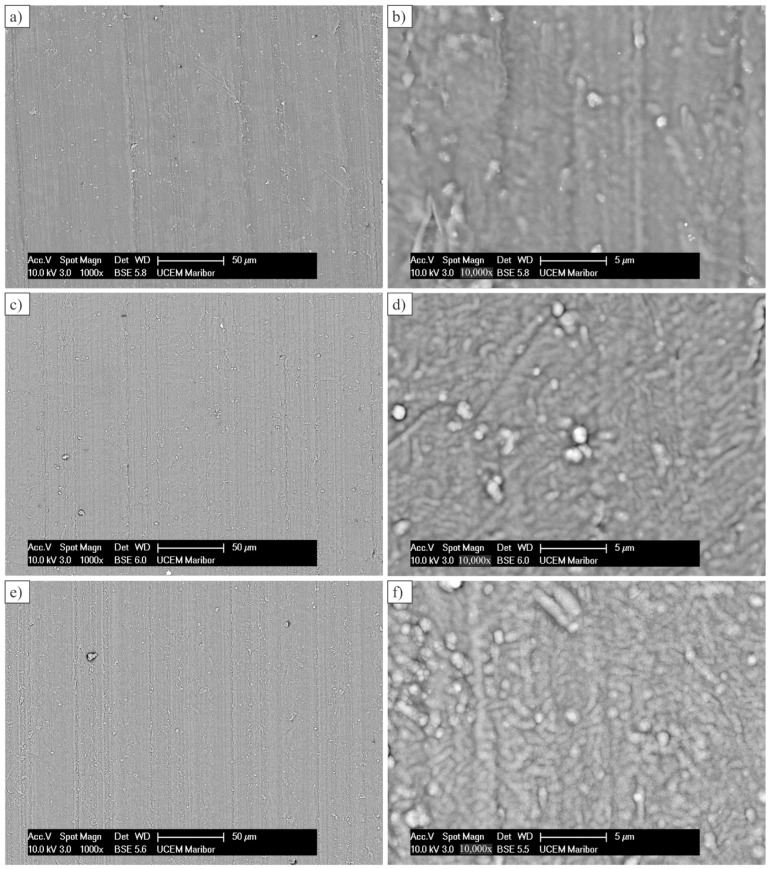
Coated gear surfaces at two magnifications: (**a**,**b**) One coating layer; (**c**,**d**) Three coating layers; (**e**,**f**) Five coating layers.

Figure 4 shows the backscattered electron images of the Al-coated gear surface. At smaller magnifications, there were no significant differences between the surface of one and three aluminium layers in comparison to the surface of the POM substrate. However, the surface with five Al layers was rougher, caused by many growth defects.

**Figure 5:**
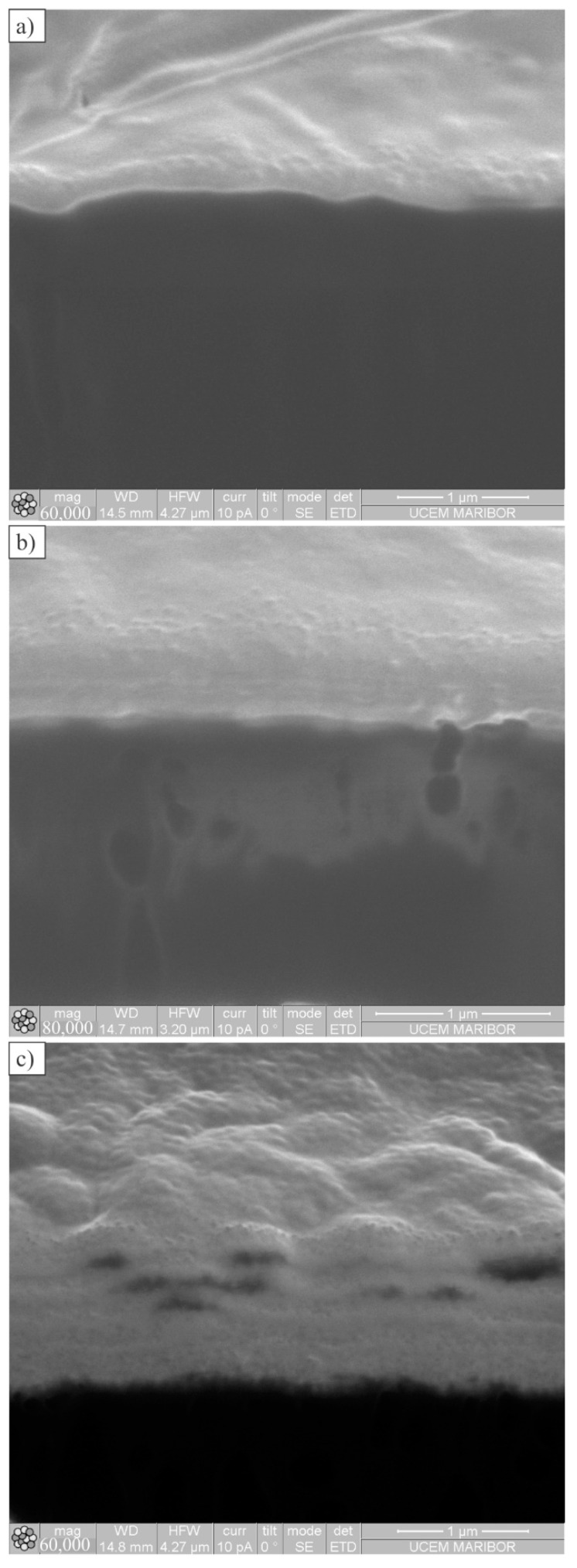
The SEM-FIB cross-sections of coated gear surfaces: (**a**) One coating layer; (**b**) Three coating layers; (**c**) Five coating layers.

Figure 5 shows the cross-sections made by SEM-FIB. The thicknesses were 301 ± 16, 657 ± 26, and 1188 ± 225 nm for one, three, and five coating layers of Al, respectively. After the deposition of each layer, the coating was subjected to plasma activation, which, in fact, removed a part of the previous layer. Thus, the total thickness of five layers is smaller than multiples of the thickness of the first layer. The single layer seems to be well attached to the POM substrate. Three layers are compact, although some interfaces are visible between the layers. In the case of five layers, some porosity appeared between the individual layers, which made the surface wavy in the submicrometre region. It is supposed that during deposition, stresses occurred between layers, causing their separation.

We carried out the microchemical analysis (EDS) at 5 kV accelerating voltage to minimise the interaction volume in order to avoid a signal from the substrate POM. The EDS showed that the layer contained 7.9–10.6 wt.% C, 6.9–13.6 wt.% O, 4.3–5.6 wt.% Si, and 71.5–79.0 wt.% Al. There were not many differences with different numbers of Al-layers. Thus, almost the whole interaction volume was in the coating, even at one Al-layer. The signals from C, O, and Si came from the outermost HMDSO protective layer. The presence of oxygen can also mean a there is a small fraction of aluminium oxide in the layer, which could act as an abrasive agent.

### 3.2. Adhesion Analysis

**Figure 6:**
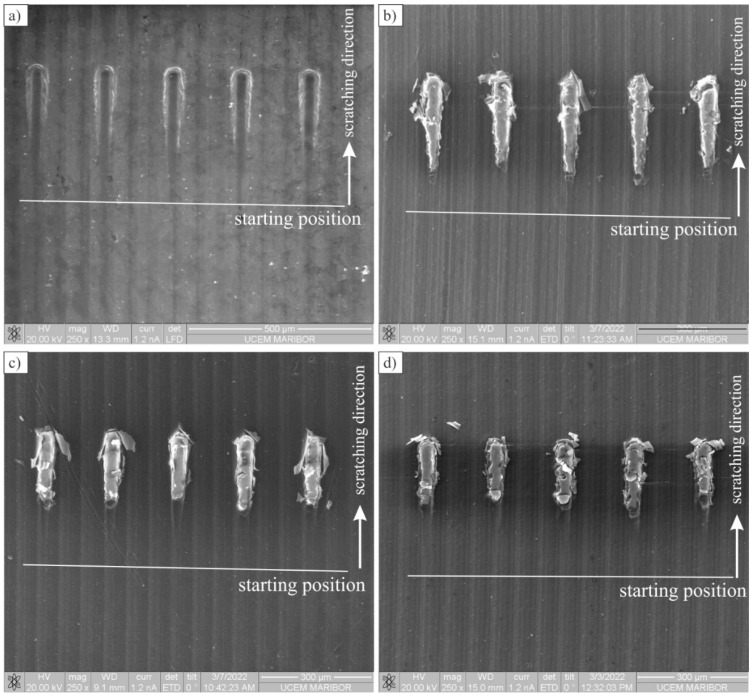
Backscattered electron micrographs of scratches (The white line indicates the position at which scratch tests started): (**a**) Uncoated POM, (**b**) One coating layer, (**c**) Three coating layers, (**d**) Five coating layers.

Figure 6 shows the scratches on the uncoated and Al-coated surfaces. The scratches on the uncoated surface were made to obtain the behaviour of the substrate material (POM) during scratching with a spherical indenter. On the coated samples, the initial damage was the formation of cracks in the coating. The length at which cracks appeared decreased with the increasing number of layers. On the micrographs, the distances were measured where the first crack was formed.

**Figure 7:**
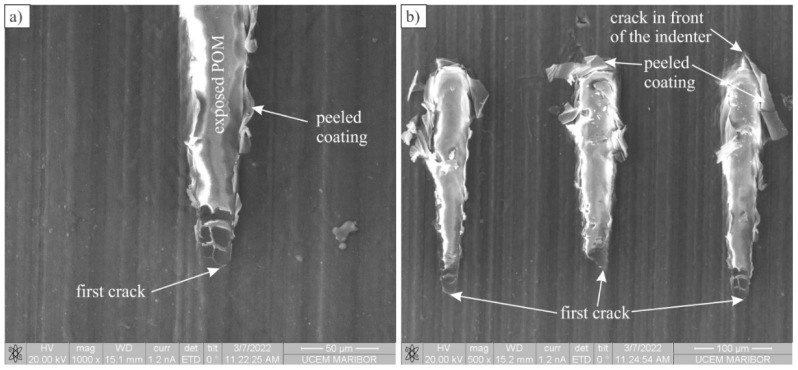
Backscattered electron micrographs of scratch tracks showing the damage: (**a**) A detailed view of the scratch beginning; (**b**) Damage caused by the indenter in the POM, coated with one layer of Al.

The average values of distances and loads, together with the standard deviations, are given in Table 5, while detailed damage is given in Figure 7. Soon after the formation of cracks, the coating collapsed, and the spherical indenter penetrated into the substrate. The following process was similar to the uncoated POM.

Typically, a crack was formed in the coating at the centre or at the edge of the indenter and then propagated in the direction of scratching. Simultaneously, the coating started to peel off the substrate, sideways or in the scratching direction. This suggests that the adherence between the substrate and coating was not sufficiently high.

**Figure 8:**
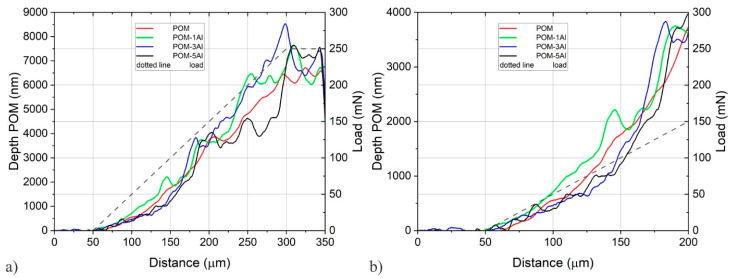
The profiles of selected scratches after scratching (load 0.1 mN): (**a**) The whole range; (**b**) Details at lower loads.

Figure 8 shows the profiles of selected scratches that were damaged at approximately the same distance as the average distance for the formation of scratches at a particular number of Al layers. For reference, the scratch profile of the uncoated POM is also shown. The elastic deformation of the uncoated POM occurred at approximately 20 mN. Afterwards, the plastic deformation increased evenly up to 125 mN (distance of 200 μm) and then a pop-in appeared. It was probably caused by the pile-up of the substrate material in front of the tip. The tip overcame the piled-up material and then penetrated further into the substrate. This appeared on several occasions, especially at higher loads. This process is usually called smearing.

It should be stressed that the profile for one coated layer is at higher depths than for the uncoated POM. It could mean that the POM is softer after deposition due to thermal exposure and that just one coated layer could not take an additional load. The first cracks on the coating occurred at approximately 50 mN (length 100 µm), but the collapse of the coating was still prevented up to the load of approximately 75 mN when the depth increased suddenly. Three and more layers support more load until the layer breaks, which was characterised by the rapid increase in depth, followed by a pop-in. The final depth was even deeper than that of the uncoated POM, which additionally suggests that the thermal exposure during coating decreases the properties of the substrate considerably.

### 3.3. Wear Behaviour of the Analysed Gears

**Figure 9:**
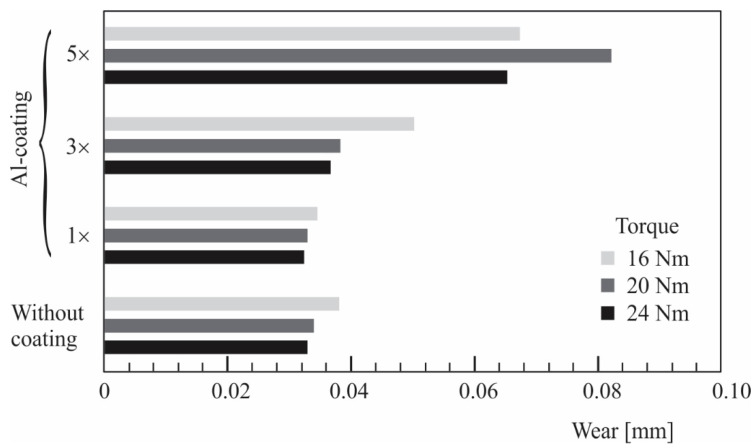
Wear of uncoated and coated polymer gears.

Figure 9 shows the wear behaviour of the uncoated and coated polymer gears at different torques of 16, 20, and 24 Nm. It can be seen that the wear of the uncoated polymer gears increased with increasing torque. Similar conclusions can also be made for the coated polymer gears considering 1, 3, or 5 coating layers. The exception was the surface coating with five layers, where the maximum wear appeared for the torque 20 Nm. This was probably due to the bad quality of the surface coating in this case. However, a negative influence of the surface coating on the wear resistance of analysed polymer gears was obtained, especially in the case of five coating layers. This phenomenon could be explained due to the fact that the surface coating was removed at a very early stage of experimental testing and then acted as an abrasive material between the meshing gear flanks. As already described in Section 3.1, some porosity also appeared between the individual coating layers, which may have led to their separation.

**Figure 10:**
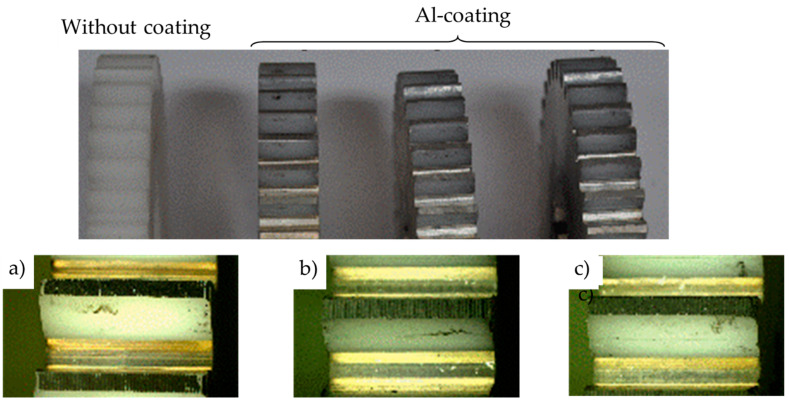
Wear of uncoated and coated polymer gears: (**a**) One coating layer; (**b**) Three coating layers; (**c**) Five coating layers.

Figure 10 shows the wear of the coated polymer gears for 1, 3, and 5 coating layers. Since the surface coating was removed at a very early stage of gear operation, the subsequent wear behaviour is similar to that applied by the uncoated gears. As already explained in Section 1, wear is a typical surface failure that usually appears by nonlubricated contact of a meshing gear pair. In addition, both sliding and rolling movements are present in the engagement of the gear teeth, which, in addition to the external load on the gear pair (torque), contributes significantly to the wear of the contact surfaces. Figure 10 also shows that the surface coating is almost completely removed from the base material due to poor adhesion of Al coatings to the POM gear surface.

When evaluating the wear behaviour of coated polymer gears, different mechanisms describe the adherence of metals to polymers: mechanical interlocking, interdiffusion, electrostatic attraction, and the adsorption theory. Typically, the first three mechanisms are rare, and if active, they are rather weak. The adsorption mechanism is the most important and can result in both physical and chemical adsorption. Al atoms can form stronger bonds when the polymer surface contains functional groups (amine or hydroxyl). POM itself does not contain any functional groups that could combine well with Al. Prior surface plasma activation in the air can produce some amine functional groups, but they were apparently rare. Plasma activation also increases polymer surface tension, which can contribute to better physical adherence.

In regard to the obtained experimental results and discussion as described above, additional investigation regarding the PVD-coating procedure should be performed in the future to improve the adhesion characteristics of the applied Al coatings and, consequently, to improve the wear behaviour of coated polymer gears.

## 4. Conclusions

An experimental investigation of the wear behaviour of coated spur polymer gears made of POM was performed in the presented study. Three different thicknesses of aluminium (Al) coatings with one, three, and five coating layers deposited by the Physical Vapour Deposition (PVD) process were investigated, considering the coating morphology, adhesion analyses, and wear behaviour. Based on the obtained experimental results, the following conclusions can be made:

- The indentation tests made on both the uncoated and coated samples have shown a very small increase in indentation hardness and modulus due to the Al-coating. However, the indentation hardness and reduced modulus increased with the increasing thickness of surface coatings and were much lower than the known properties of pure aluminium.
- The SEM analysis of the coated surfaces has shown that the surface with the five Al-layers was rougher compared to surfaces with one or three coating layers. Furthermore, some porosity appeared in the surface coating with five coating layers, which probably arose during the deposition process and may have led to the separation of the individual layers.
- The comprehensive adhesion analysis has shown that the adherence between the substrate (POM) and Al coating was not sufficiently high, which may have led to the separation of the coated surface layer in a very early stage of gear operation.
- For both the uncoated and coated polymer gears, the wear increased with increasing torque. However, a negative influence of the surface coating on the wear resistance of analysed polymer gears was obtained, especially in the case of five coating layers. This phenomenon could be explained due to the fact that the surface coating was removed at a very early stage of experimental testing and then acted as an abrasive material between the meshing gear flanks.
- Based on the general findings as explained above, it can be concluded that the influence of the analysed Al coatings on the wear behaviour of POM polymer spur gears is small and does not reduce the wear of meshing gear flanks. For that reason, a systematic investigation of the complete PVD procedure is recommended in the future to improve the adhesion characteristics between a POM substrate and an Al coating. Furthermore, the other PVD coatings (Cr, CrN, etc.) may be considered in further research work related to the wear behaviour of coated polymer gears.
- For better adhesion of aluminium to polymer samples, it would be reasonable to optimize the plasma activation process. Parameters that could possibly improve adhesion are a higher power, longer process step, use of argon instead of air, etc. Finer surface treatment of the polymer samples and degreasing the samples before the sputtering process might also help to improve adhesion.

## Figures and Tables

**Table 1:** Basic mechanical, thermal, and physical properties of the analysed POM (Adapted from [53], Faigle, 2022).

Mechanical Characteristics	Standard	Unit	Value
Yield stress (+23 °C, dry)	ISO 527-1/-2	MPa (N/mm^2^)	67
	DIN 53455		
	ASTM D 638		
Tensile strength (+23 °C, dry)	ISO 527-1/-2	MPa (N/mm^2^)	66
	DIN 53455		
	ASTM D 638		
Elongation at break (+23 °C, dry)	ISO 527-1/-2	%	40
	DIN 53455		
	ASTM D 638		
Tensile *E*-modulus (+23 °C, dry)	ISO 527-1/-2	MPa (N/mm^2^)	2800
	DIN 53455		
	ASTM D 638		
Charpy notched impact strength (+23 °C, dry)	ISO 179	kJ/m^2^	6
	DIN 53453		
Ball indentation hardness (dry)	ISO 2039-1	MPa (N/mm^2^)	130
Thermal characteristics	Standard	Unit	Value
min. operating temperature (continuous)	-	°C	−50
max. service temperature (continuous)	-	°C	100
max. service temperature (short-term)	-	°C	140
Thermal conductivity (+23 °C)	DIN 52612	W/(m × K)	0.31
Physical characteristics	Standard	Unit	Value
Density	ISO 1183	g/cm^3^	1.41
	DIN 53479		
	ASTM D 792		
Moisture absorption at saturation (23 °C/50% r.h.)	ISO 62 ISO 1110	%	0.20

**Table 2:** Process parameters for one layer of aluminium PVD coating.

Process	Pumping Time (s)	Starting Pressure (mbar)	Mass Flow Contr. MFC (sccm)	Regulation Pressure (mbar)	Process Time (s)	Regulation Energy (kWs)	*T* (°C)	
							min	max
Plasma activation	10	5·10^−3^	800	3·10^−2^	18	198	500	5000
Magnetron sputtering	150	4·10^−4^	500	2.2·10^−3^	62	10500	30	90
Plasma polymerisation	1	1.5·10^−2^	300	2·10^−2^	50	582	500	5000

**Table 3:** Basic parameters of the tested gear pair.

Parameter	Tested Gear	Supported Gear
Material	POM	Steel (16MnCr5)
Normal module *m*	2.5 mm	2.5 mm
Pressure angle α_n_	20°	
Helix angle β	0°	
Number of teeth *z*	36	36
Tooth width *b*	14 mm	14 mm
Profile shift coefficient *x*	0	
Centre distance a	90 mm	
Basic rack profile	ISO 53	
Lubrication	Dry (not lubricated)	

**Table 4:** Indentation hardness and modulus in the range between 1 and 5 mN.

Surface Layer	Indentation Hardness (MPa)	Indentation Reduced Modulus (GPa)
Al 99.99	376.5 ± 13.2	69.16 ± 3.1
POM	100.0 ± 0.2	3.16 ± 0.20
POM-1 layer Al	172.1 ± 33.3	5.23 ± 0.61
POM-3 layers Al	214.4 ± 26.0	5.15 ± 1.08
POM- 5 layers Al	318.1 ± 62.6	5.96 ± 1.21

**Table 5:** The distances and loads at which the first cracks were observed on the coating.

Sample	Distance/µm	Critical Load/mN
One coating layer	94.2 ± 12.03	44.2 ± 12.0
Three coating layers	155.8 ± 12.7	102.8 ± 12.7
Five coating layers	186.8 ± 21.3	136.8 ± 21.3

## Data Availability

The data presented in this study are available on request from the corresponding author.
